# Variation in the Performance of Different Batches of Two *Mycobacterium avium* Subspecies *paratuberculosis* Antibody ELISAs Used for Pooled Milk Samples

**DOI:** 10.3390/ani12040442

**Published:** 2022-02-12

**Authors:** Heike Köhler, Annika Wichert, Karsten Donat

**Affiliations:** 1 Institute of Molecular Pathogenesis, Friedrich-Loeffler-Institut, Federal Research Institute of Animal Health, Naumburger Strasse 96a, 07743 Jena, Germany; 2 Thuringian Animal Diseases Fund, Victor-Goerttler-Strasse 4, 07745 Jena, Germany; awichert@thtsk.de (A.W.); kdonat@thtsk.de (K.D.)

**Keywords:** enzyme linked immunosorbent assay (ELISA), *Mycobacterium avium* subspecies *paratuberculosis* (MAP), S/P value, receiver operating characteristic (ROC) analysis, batch differences, pooled milk samples

## Abstract

**Simple Summary:**

This article explores variation in the performance of different batches of tests for the detection of antibodies against the ruminant pathogen *Mycobacterium avium* subspecies *paratuberculosis* (MAP) in milk. The results indicate that variation is present and that it has sources mainly in the manufacturing process of the test kits and, to a lesser degree, in the test laboratories.

**Abstract:**

Regionally, the monitoring of paratuberculosis at the herd level is performed by the detection of specific antibodies in pooled milk samples by ELISA. The negative/positive cut-off S/P values applied for pooled milk samples are low and particularly vulnerable to variation in the test performance. In this study, a batch variation in the test performance of two ELISA tests was assessed to identify consequences for sample classification. A total of 72 pooled milk samples (50 from MAP-infected herds, 22 from one MAP-non-infected herd) were analyzed using three different batches, each of two different MAP antibody ELISA tests (A and B). Receiver operating characteristic (ROC) analysis was performed, with the results of each batch, S/P values of the samples and optical density (OD) readings of the negative and positive control samples included in the kits being compared between the batches of one test. ROC analysis revealed a considerable variation in the test performance of the batches of the two individual tests, caused by differences in the S/P values of the samples and resulting in different sensitivities at a specificity of 100%. Major sources of variation originate from the manufacturing processes of test batches. These sources have to be better controlled, and the test performance has to be revisited regularly.

## 1. Introduction

Paratuberculosis is a worldwide-spread disease that causes substantial financial losses in affected dairy herds [1,2]. The causative organism of the disease is *Mycobacterium avium* subsp. *paratuberculosis* (MAP). To identify MAP-infected animals or herds, direct and indirect methods of pathogen detection are applied. Common diagnostic tools used for the direct diagnostics of MAP are bacterial culture and PCR [3]. Paratuberculosis can also be diagnosed indirectly by the detection of MAP-specific antibodies in serum samples or milk using ELISA [4,5].

Especially in the context of paratuberculosis control programs, diagnostics based on milk ELISA have several advantages compared with other testing strategies. Firstly, ELISA testing is less time consuming than bacterial culture. Due to the long replication time [6], 16 weeks of cultivation are recommended [7], whereas ELISA results are available within one day. Secondly, individual milk or bulk milk samples taken regularly at the monthly dairy herd improvement testing are easily available test materials. To obtain these samples, no additional handling of animals is necessary. Thirdly, testing pooled milk samples or bulk milk by ELISA is a low-cost MAP-surveillance strategy [8], which is efficient in the detection of MAP-infected herds with a serological within-herd prevalence (ELISA-positive results as a share of all tested serum samples) of at least 8%. A German study showed that these herds can be detected with a probability of 95% using milk pools [9]. Meanwhile, this strategy is applied in paratuberculosis control programs. In the German federal state Lower Saxony, for example, a mandatory program for the reduction of MAP infections in cattle herds was implemented, where the analysis of milk pools of size ≤50 by ELISA is utilized for the surveillance of MAP-infected dairy herds [10].

If milk serology is used to detect MAP-infected herds, two further aspects have to be considered. First, the specificity of milk ELISAs is reduced compared to direct MAP detection methods and ranges between 83% and 100% [11]. Second, the sensitivity of ELISA tests is lower than the sensitivity of the bacterial culture or PCR [12]. Furthermore, the sensitivity of an ELISA test is even lower if pooled milk samples are used instead of individual milk samples due to the dilution effect occurring when pooling samples [13]. Therefore, the cut-off value has to be adapted for pooled samples [13,14]. For the commercial ELISA test kits that are accredited for MAP-diagnostics in Germany, adapted cut-offs for milk pools have been recommended based on receiver operating characteristic (ROC) analysis [14].

For serum ELISAs, a high variation in the test performance between different batches of commercial ELISA kits was reported [15]. If this also applies for milk ELISAs, this may have implications for the classification of samples, especially with S/P values near the cut-off, and, consequentially, for the assessment of the herd status when pooled or bulk milk testing is applied for surveillance.

## 2. Materials and Methods

In the present study, we analyzed the variation in test performance of different batches of two commercial ELISA tests (test A and B) for the detection of antibodies against MAP with a set of 72 milk pools. These pools had been used previously for the definition of cut-off values of these ELISAs for pool-milk testing. The pools were prepared essentially as described elsewhere [14]. In brief, individual milk samples taken for monthly dairy herd improvement testing were derived from two MAP-infected and one MAP non-suspect dairy cattle herds. The MAP status of the study herds and the cows whose milk was pooled was known from individual fecal culture as well as from individual milk and serum ELISA testing. The milk samples were de-fatted after centrifugation and the milk serum was stored frozen until further processing. Pools of 50 individual samples were prepared separately for each herd and sampling day. Therefore, equal volumes (100 µL) of randomly selected individual samples were pooled in such a way that each sample was used only once per sampling and that the animal cohorts that contributed to the individual pools varied between sampling days. Pooling resulted in a total of 72 pools: 50 from MAP-infected herds and 22 from the MAP non-suspect herd [14]. For both ELISA tests, short incubation protocols for milk samples were performed, comprising pre-incubation of the samples with a *Mycobacterium phlei*-suspension (provided with the kit) for 15 min at room temperature followed by incubation of the pre-treated samples on the ELISA plates for 45 min at room temperature. Further steps were performed essentially according to the instructions of the manufacturers. Negative and positive control samples provided with the kits were run in duplicate on each ELISA plate. To control for intra-plate variation, the pooled milk samples were also run in duplicate, and mean OD values were calculated. The ELISA tests were performed by the same experienced laboratory technician as in the preceding study.

Sample-to-positive ratio (S/P) values were calculated, essentially according to the instructions of the manufacturers. The test performance of two recent batches, each of test A (I and II) and test B (I and II), was compared to the test performance of the batches that had been applied for cut-off definition in the preceding study (batch A-0 and B-0) [14]. The milk pools from the MAP non-suspect herd were classified as negative and the pools from the two MAP-infected herds were classified as positive, even if only animals with negative contemporaneous individual milk and serum antibody test results, as well as negative fecal culture results, contributed to the pool [14]. ROC analysis was performed using MedCalc Statistical Software version 14.8.1 [16]. The area under the curve (AUC), the difference between areas and the significance levels of these differences (P) were assessed. If the resulting *p*-value was *p* ≤ 0.05, statistical significance was assumed. For each batch, the cut-off S/P value was selected in a way that maximum test sensitivity was achieved given a test specificity of 100%. Variation in the test results was visualized by plotting the S/P values of the three batches of each test in one graph, arranging the samples in the order of magnitude of the S/P values resulting from batch A-0 and B-0, respectively [17]. The optical density (OD) values of the positive control samples included in kits of different batches were compared using the Mann–Whitney U-test. The OD values of the negative control samples were compared in the same way.

## 3. Results

**Figure 1:**
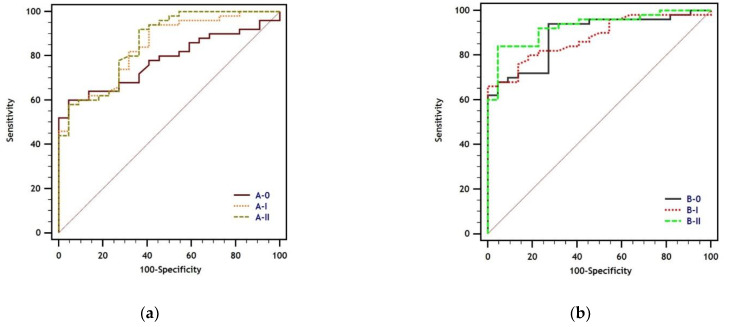
Receiver operating characteristic (ROC) curves of three different batches, each of (**a**) test A and (**b**) test B.

ROC analysis unveiled differences in the test performance of different batches of both ELISA tests, which were more pronounced for test B, but did not result in significant differences in the AUC (Figure 1, Table 1). A specificity of 100% was reached at individual cut-off values for each batch (Table 2). Given a specificity of 100%, the sensitivity was reduced in batches A-I and A-II compared to batch A-0. Regarding test B, the sensitivity varied between the batches at a specificity of 100% (Table 2).

**Figure 2:**
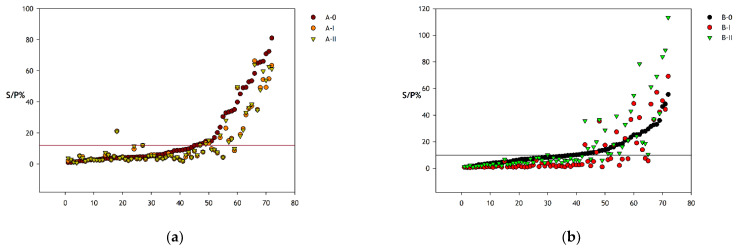
Distribution of sample-to-positive ratio (S/P) values of different batches of (**a**) ELISA test A and (**b**) ELISA test B. The samples were arranged in the order of magnitude of their S/P values resulting from testing with either batch A-0 or batch B-0. Labels on the x-axis represent the ranking order of these samples according to S/P value in batch 0. Horizontal lines indicate the cut-off S/P-values calculated for batch 0 of the respective test.

The S/P values of batch A-I and A-II did not differ markedly but tended to be lower than those of batch A-0, particularly for samples with S/P values around and above the cut-off value recommended for test A in the preceding study [14] (Figure 2A). The S/P values of all three batches of test B differed considerably. Samples with low S/P values using batch B-0 reached even lower, and, again, reached different S/P values using batch B-I and B-II, respectively. The variation between batch B-I and B-II was most pronounced and random in samples with S/P values around and above the cut-off value recommended for batch B-0 in the preceding study [14] (Figure 2B).

The OD values of the positive control samples of batch A-I were significantly higher than the OD values of batch A-0 and A-II (Mann–Whitney U-test, *p* = 0.01 and 0.029, Table 3). In contrast, the OD values of the negative control samples did not differ markedly between the three batches of ELISA test A (Mann–Whitney U-test, *p* = 0.476−0.486). The differences between the positive control samples of test B were even more pronounced (Mann–Whitney U-test, *p* = 0.029). The OD values of the negative control samples of the recent batches of test B were lower than those of batch B-0, the difference being significant for batch B-I (Mann–Whitney U-test, *p* = 0.029, Table 3).

## 4. Discussion

The present data reveal variations in the performance of different batches of paratuberculosis antibody ELISA tests. These variations have implications for the assessment of pooled milk samples. In order to account for these variations, in theory, different cut-off values have to be applied for every batch. Due to the fact that this is not practicable, some uncertainty exists regarding the assessment of samples with S/P values around the recommended cut-off value. Test manufacturers tackle uncertainty in the classification of individual serum and milk samples by introducing a range of S/P values classifying samples as non-conclusive. In such cases, a re-testing of the sample or repeated sampling of the same animal is recommended to verify the result. As the cut-off values suggested for the pooled milk samples are rather low [14,18], a non-conclusive range is not applicable. Both the sensitivity and specificity of the ELISAs for pooled milk samples would be reduced.

A number of factors have to be considered as reasons for the variation in the test performance of different batches of the same ELISA test seen in this study. The time gap of approximately five years between the testing of batches A-0 and B-0 in the preceding study [14] and the testing of batches A-I, A-II, B-I and B-II may have resulted in changes in the laboratory environment and calibration of instruments. These factors are all considered as controllable sources of variation [15]. They were kept as stable as possible when testing the recent batches. All testing was carried out by one very experienced laboratory technician. Furthermore, the samples were stored frozen for the time period between the preceding and the present study.

It was not possible for us to control the effects of the long-term storage of the pooled milk samples for approximately five years on the variability of the S/P values. Therefore, such effects cannot completely be neglected. The freezing of bulk milk samples for 25–28 days at −20 °C led to an overestimation of the percent positivity values of 0.4 percentage points compared to fresh samples using an ELISA for antibodies against *Salmonella* Dublin [19]. The results of others, however, indicate that stressors, such as freezing for up to 8 months, thawing and re-freezing, result in biologically negligible differences in the S/P values or OD ratios [20,21]. In our study, the fact that the individual samples react non-uniformly when tested with the recent batches of the two different ELISA tests underlines that other sources of variability have to be considered too.

Sources of variation that are not controllable by the user of the test kit are the consistency of the assay reagents, including negative and positive control samples [15], and the composition of the antigen batches that are used for the coating of the ELISA plates [21]. The latter applies, in particular, when heterogeneous mixtures of antigens are used, such as the protoplasmic antigen preparations from MAP cultures that are used in ELISA A and B. Standardization of bulk cultures of MAP is difficult. Consequentially, it has to be expected that the composition of different antigen preparations is not completely identical and that the amounts of individual antigens vary. Positive milk samples contain variable proportions of antibodies with different antigen specificities. Their test response will depend on how much of the reacting antigen is coated to the plate [21]. It is very likely that the antigen batches used for coating the ELISA plates of both tests have changed within the time period of five years. This may have contributed to the lower S/P values of antibody-positive samples obtained with batch A-I and A-II. The variability of the test results of antibody-positive samples between the batches of ELISA test B might also be due to the different compositions of the antigen preparations used.

The OD values of the negative and positive control samples are included in the calculation of the S/P values. Their reactivity is adjusted during the quality assurance process implemented by the manufacturers for each test batch to control for the variability of antigen coating and assay reagents using well defined sera as calibrators. The variation in their adjustment results in a variation in the calculated S/P values of test samples. Samples with S/P values around the cut-off are most affected because they might be classified as negative using one batch and as positive using another. It is conceivable that either the control samples, the calibrators or both have changed between the batch used in the preceding study and the recent batches. The mean OD values of the positive control samples varied considerably between the batches of both ELISA tests. These OD values of the two recent batches of each test were higher than the values of the batch used in the preceding study. This may have contributed to the lower S/P values of positive samples achieved with batch A-I and, to a lesser degree, A-II, and, likewise, of negative samples achieved with batch B-I and B-II. On the other hand, despite significant differences between the mean OD values of the positive control samples of batch A-I and A-II, the reactivity of these samples seemed to be adjusted in a way that similar S/P values were obtained for the tested samples. The positive and negative control samples of the batches of ELISA B, however, were not adjusted accordingly, resulting in divergent S/P values of the samples. 

Altogether, we assume that a combination of different sources contributes to the variation in the test performance of different batches of the same ELISA test. The relative impact of these sources is difficult to quantify. Great demands are made to the quality assurance protocols during the manufacturing processes of the assays. High standards have to be applied to antigen preparation and coating. A careful standardization of the calibrator sera and proper adjustment of positive and negative control samples are essential. Despite appropriate efforts of the manufacturers, a variation in the test performance over time seems to be inevitable. Therefore, users are advised to re-examine the test performance regularly with a panel of well-defined field samples. 

Pooled milk serology is considered to be the least sensitive approach for the herd level diagnosis of paratuberculosis [14], limiting its diagnostic value in paratuberculosis control programs. Nonetheless, it can be the first step to identify the most affected herds in regions with a high paratuberculosis prevalence at the herd level. Thus, differences in the test sensitivity of different ELISA batches increase the uncertainty of this diagnostic approach This underlines that ELISA tests applied on pooled milk serology should be of high homogeneity with well-defined and proper adjusted positive and negative control samples to prevent discreditation of this diagnostic approach by the variation in test performance.

## 5. Conclusions

Variation in the performance of ELISA tests for antibodies against MAP is due to sources that can be controlled by the laboratory and sources originating in the manufacturing process of the kit batches. This variation has consequences for the test sensitivity because it results in different classifications of samples with S/P values around the cut-off value. In the case of pooled milk samples, this is of particular importance, because a non-conclusive range is not applicable for classification, as the cut-off values are generally low. During the manufacturing process, greater efforts have to be undertaken to control critical factors, such as antigen preparation, the coating of ELISA plates, the selection of calibrator sera and the proper adjustment of positive and negative control samples. Nonetheless, diagnostic laboratories should re-examine the test performance regularly with a panel of well-defined field samples.

## Figures and Tables

**Table 1:** Results of the receiver operating characteristic (ROC) analysis of different batches of two different ELISA tests (A and B) for the analysis of antibodies against *Mycobacterium avium* subsp. *paratuberculosis* in pooled milk samples.

	ROC	Pairwise Comparison of ROC Curves			
Test-Batch	AUC ^1^ (95% CI ^2^)	Difference between Areas (95% CI)		Significance Level (P)	
		A/B-I	A/B-II	A/B-I	A/B-II
A-0	0.779 (0.666–0.868)	0.064 (−0.027–0.156)	0.079 (−0.017–0.174)	0.170	0.107
A-I	0.843 (0.738–0.918)	-	0.015 (−0.012–0.041)	-	0.281
A-II	0.858 (0.755–0.929)	-	-	-	-
B-0	0.889 (0.793–0.951)	0.011 (−0.046–0.068)	0.038 (−0.005–0.082)	0.709	0.085
B-I	0.878 (0.780–0.943)	-	0.049 (−0.003–0.101)	-	0.063
B-II	0.927 (0.841–0.975)	-	-	-	-

^1^ Area under the curve, ^2^ Confidence interval.

**Table 2:** Cut-off value and sensitivity with 95% confidence intervals (CI) of different batches of two different ELISA tests (A and B) for the analysis of antibodies against *Mycobacterium avium* subsp. *paratuberculosis* in pooled milk samples at specificity of 100%.

		Test Characteristics	
Test-Batch	Cut-Off Value (S/P% ^1^)	% Sensitivity (95% CI)	% Specificity (95% CI)
A-0	12.29	52.0 (37.4–66.3)	100.0 (84.6–100.0)
A-I	9.65	46.0 (31.8–60.7)	100.0 (84.6–100.0)
A-II	11.61	44.0 (30.0–58.7)	100.0 (84.6–100.0)
B-0	10.33	62.0 (47.2–75.3)	100.0 (84.6–100.0)
B-I	2.65	66.0 (51.2–78.8)	100.0 (84.6–100.0)
B-II	8.06	60.0 (45.2–73.6)	100.0 (84.6–100.0)

^1^ Percent sample to positive ratio.

**Table 3:** Optical density (OD) readings of the batch-related positive and negative control samples of different batches of ELISA test A and B. Mean (MW) and standard deviation (SD) of duplicate readings on two ELISA plates per batch.

	ELISA A		ELISA B	
	MW (SD)		MW (SD)	
Control	PC ^1^	NC ^2^	PC	NC
Batch 0	0.807 (0.114) ^a^	0.044 (0.005)	0.433 (0.011) ^a,b^	0.062 (0.004) ^a^
Batch I	1.544 (0.340) ^c^	0.046 (0.002)	1.870 (0.257) ^c^	0.052 (0.002)
Batch II	0.932 (0.023)	0.047 (0.001)	0.996 (0.084)	0.056 (0.010)

^1^ Positive control sample included in the respective batch, ^2^ negative control sample included in the respective batch, ^a,b,c^ statistically significant differences between the respective positive or negative controls of batches of the same ELISA test, ^a^ between batch 0 and I, ^b^ between batch 0 and II, ^c^ between batch I and II, Mann–Whitney U-test, *p* ≤ 0.05.

## Data Availability

The data presented in this study are available on request from the corresponding author. The data are not publicly available due to confidentiality that has to be kept for data that are generated in the course of batch release testing of diagnostic tests.
